# A simulation study to investigate an extension to the point cluster technique

**DOI:** 10.1038/s41598-023-47144-2

**Published:** 2023-11-15

**Authors:** Vivek Karmarkar, Rachel V. Vitali

**Affiliations:** University of Iowa, Mechanical Engineering, Iowa City, 52242 USA

**Keywords:** Mechanical engineering, Biomedical engineering

## Abstract

Joint kinematics are an important and widely utilized metric in quantitative human movement analysis. Typically, trajectory data for skin-mounted markers are collected using stereophotogrammetry, sometimes referred to as optical motion capture, and processed using various mathematical models to estimate joint kinematics (e.g., angles). Among the various sources of noise in optical motion capture data, soft tissue artifacts (STAs) remain a critical source of error. This study investigates the performance of the point cluster technique (PCT), an extension of the PCT using perturbation theory (PCT-PT), and singular value decomposition least squares (SVD-LS) method (as a reference) for 100 different marker configurations on the thigh and shank during treadmill walking. This study provides additional evidence that the PCT method is significantly limited by the underlying mathematical constraints governing its optimization process. Furthermore, the results suggest the PCT-PT method outperforms the PCT method across all performance metrics for both body segments during the entire gait cycle. For position-based metrics, the PCT-PT method provides better estimates than the SVD-LS method for the thigh during majority of the stance phase and provides comparable estimates for the shank during the entire gait cycle. For knee angle estimates, the PCT-PT method provides equivalent results as the SVD-LS method.

## Introduction

Joint kinematics, defined as the relative motion between two adjacent bones, are an important and widely utilized metric in quantitative human movement analysis^1^. Most commonly, retroreflective markers are attached to body segments on the skin whose positions are triangulated via infrared cameras (i.e., stereophotogrammetry)^2^. These skin-mounted markers are inherently susceptible to a critical source of noise – soft tissue artifacts (STAs)^3–6^, or the relative motion between the skin-mounted markers and the underlying skeletal structure^7^. Since naive models cannot account for this type of noise^8^, the skin-mounted marker data are processed using a mathematical model to extract the desired information, which are sometimes referred to as bone pose estimators (note that pose refers to position and orientation). Past research shows STAs have a frequency content similar to actual bone movement, are task dependent, are subject specific, and are greatest in magnitude at the thigh^9^. Furthermore, STAs can be represented as a combination of rigid body (i.e., translation and rotation) and non-rigid body (i.e., scaling and deformation) components^10^. The literature details numerous methods designed to mitigate the substantial effects of STAs including the “solidification” procedure^11^, multiple anatomical landmark calibrations^12^, pliant surface modeling^13^, dynamic calibrations^14^, the point cluster technique (PCT)^15^, and the singular value decomposition least squares (SVD-LS) pose estimator^16^. Note that many of these methods are better suited to address the non-rigid body components of STA while others^17^ are designed to address the rigid body components of STA.

The SVD-LS pose estimator is utilized extensively in the literature as a robust approach to mitigate largely non-rigid modes of STA from raw marker data. This method relies on the singular value decomposition^18^ to solve a least squares fitting problem for two three-dimensional sets of points at each time step. This results in estimates of the rotation matrix (orientation) and position vector (translation) at each time step for the body segment. Cereatti et al.^19^ performed an extensive comparative assessment of different bone pose estimators including SVD-LS, a geometrical method, unoptimized PCT, and optimized PCT. The estimators’ performance in estimating knee kinematics was evaluated in presence of STAs as well as instrumental noise and anatomical landmark mislocations for the thigh segment. Overall, it was found that the SVD-LS bone pose estimator yielded superior results, which in turn led to its widespread use today.

A common feature of the aforementioned methods is each body segment is treated independently^9^. However, the absence of biomechanical constraints can sometimes yield physiologically meaningless joint kinematics^9^ (for example, joint translations ranging from 12 mm to 29 mm have been reported by^20^). Global optimization methods^21^ have been proposed as an alternative approach belonging to the multi-body kinematics optimization family of methods^22^, some of which have been found to be unreliable solutions to mitigating STAs^23^. This method incorporates joint constraints in a cost function via a mathematical model describing a ball-and-socket joint, which means this method is somewhat limited by treating *every* joint as a ball-and-socket^9^. For example, Lu et al.^21^ utilized a sinusoidal function proposed by Cheze et al.^11^ to artificially represent STAs in the evaluation of their multi-link musculoskeletal model that exploits this ball-and-socket assumption. While^21^ showed minimal errors in axial rotation as well as abduction/adduction rotations at the joints, a subsequent validation study that used dual-plane fluoroscopy as ground truth revealed significant errors (i.e., mean errors of 10 degrees for rotations and 10-15 mm for translations)^24^.

Unlike the SVD-LS and global optimization methods, the PCT is grounded in physics *and* is free of potentially controversial joint constraint assumptions. The PCT assumes a body segment is a rigid body, which means the body segment’s moment of inertia in a body-fixed frame of reference must be constant. Assuming each marker to have unit mass, the eigenvectors, eigenvalues, and center of mass (CM) of the subsequent mass distribution are calculated at each time step. Then, the mass of each marker is systematically adjusted such that the difference between norm of current time step’s eigenvalues and those of a reference (i.e., those from a static calibration) is minimized. The segment pose is then given by the eigenvectors and CM of the newly adjusted mass redistribution. Note that this method is specifically designed to address non-rigid body STA components.

Importantly, details regarding how to optimize the algorithm’s performance are not well understood. For example, an important assumption underlying the PCT is a uniform marker distribution, but the characteristics governing the nature of the uniform marker distribution (e.g., minimum marker separation) are presently unknown^15^. While this method is theoretically compatible with N> 3 markers, the proof-of-concept study demonstrated the validity for $N = 8$ markers only^15^. To address the fact that the PCT tends to fail in cases where marker clusters possess axes of rotational symmetry^15^, Carman et al.^25^ carried out a more extensive investigation into marker configurations that caused the PCT to fail during movement trials. The PCT results suggested that anatomical frame axis alignment agreed very well with the SVD-LS results for the same settings. However, this study did not have ground truth data for comparison, used a similar $N = 8$ marker configuration^15^, and only considered the shank – a body segment known to have minimal STAs. As aforementioned, Cereatti et al.^19^ conducted a comparative assessment of the performance of various bone-pose estimators for a typical flexion-extension pattern during a gait cycle, which was corrupted with instrumental noise and STAs. Two marker configurations ($N = 4$ and $N = 12$ markers) were used for both the thigh and shank. Under their experimental conditions, the PCT failed to produce any results for $N = 4$ markers and produced inconsistent results for $N = 12$ markers. The authors concluded that these findings were a result of the PCT algorithm optimizing the eigenvalue norm as opposed to individual eigenvalues themselves^19^.

While the PCT holds promise, past research also provides clear evidence of the dependence of the PCT’s performance on the number and configuration of markers. Thus, the purpose of this study is twofold. First, this study describes and evaluates a novel bone pose estimation method that extends PCT by utilizing perturbation theory, optimizing *individual* eigenvalues, and using a mass redistribution approach that is independent of analytical constraints. Next, a comparative assessment of the traditional PCT, the SVD-LS, and the novel methods for various marker configurations is conducted.

## Methods

### PCT with perturbation theory

Assuming the markers have unit mass, the inertia tensor, $\pmb {I}(t)$, is given by Eqn. (1):

1 $$\begin{aligned} I(t) = \begin{bmatrix} \displaystyle \sum _{j=1}^{N} {y_{j}(t)}^2 + {z_{j}(t)}^2 &{} -\displaystyle \sum _{j=1}^{N} x_{j}(t)y_{j}(t) &{} -\displaystyle \sum _{j=1}^{N} x_{j}(t)z_{j}(t) \\ -\displaystyle \sum _{j=1}^{N} x_{j}(t)y_{j}(t) &{} \displaystyle \sum _{j=1}^{N} {x_{j}(t)}^2 + {z_{j}(t)}^2 &{} -\displaystyle \sum _{j=1}^{N} y_{j}(t)z_{j}(t) \\ -\displaystyle \sum _{j=1}^{N} x_{j}(t)z_{j}(t) &{} -\displaystyle \sum _{j=1}^{N} y_{j}(t)z_{j}(t) &{} \displaystyle \sum _{j=1}^{N} {x_{j}(t)}^2 + {y_{j}(t)}^2 \end{bmatrix} \end{aligned}$$

where $x_j(t)$, $y_j(t)$, and $z_j(t)$ is the three-dimensional global position of the *j*th marker. Let $\pmb {\lambda }(t) = [\lambda _{1}(t), \lambda _{2}(t), \lambda _{3}(t)]^\intercal$ denote the eigenvalues and $\pmb {E}(t) = [\pmb {e}_1(t), \pmb {e}_2(t), \pmb {e}_3(t)]$ denote the corresponding eigenvectors associated with $\pmb {I}(t)$. In the presence of STAs, $\pmb {I}(t)$ and its corresponding eigendecomposition, $\pmb {\lambda }(t)$ and $\pmb {E}(t)$, are time varying. Eigenvalue perturbation theory^26,27^ can effectively “denoise” a matrix like the inertia tensor when implemented iteratively. Let $\pmb {I}_{0}$ represent the inertia tensor and $\pmb {\lambda }_{0}$ represent its eigenvalues at the initial time step, $t_{0}$, which are equivalent to those calculated from a static calibration. Since the inertia tensor is necessarily symmetric, a perturbation matrix, ${{\delta }\pmb {I}}_C$, can be defined using Eqn. (2):

2 $$\begin{aligned} {{\delta }\pmb {I}}_C = \begin{bmatrix} k_1 &{} k_2 &{} k_3 \\ k_2 &{} k_4 &{} k_5 \\ k_3 &{} k_5 &{} k_6 \end{bmatrix} \end{aligned}$$

The change in the current *j*th eigenvalue, ${\delta \pmb {\lambda }}_{C,j}$, can be computed from perturbation theory^26,27^ via Eqn. (3):

3 $$\begin{aligned} {\delta \lambda }_{C,j} = {{\pmb {e}_{C,j}}^T}{{{\delta }\pmb {I}}_C}{\pmb {e}_{C,j}} \end{aligned}$$

where $\pmb {e}_{C,j}$ is the current *j*th eigenvector. As detailed in "Appendix A" in the Supplementary Information, expanding the right hand side of Eqn. (3), multiplying the terms out, applying the result to each eigenvalue, and rearranging yields Eqn. (4):

4 $$\begin{aligned} \pmb {A}_{C}\cdot {{\delta }\pmb {K}}_C = \begin{bmatrix} ({\pmb {e}_{C,1}}^x)^2 &{} \ldots &{} ({\pmb {e}_{C,1}}^z)^2 \\ \vdots &{} \ddots &{} \vdots \\ ({\pmb {e}_{C,3}}^x)^2 &{} \ldots &{} ({\pmb {e}_{C,3}}^z)^2 \end{bmatrix} \begin{bmatrix} k_1 \\ \vdots \\ \vdots \\ k_6 \end{bmatrix} = \begin{bmatrix} {\delta \lambda }_{C,1} \\ {\delta \lambda }_{C,2} \\ {\delta \lambda }_{C,3} \end{bmatrix} = {\delta \pmb {\lambda }}_C \end{aligned}$$

The current perturbation vector, ${{\delta }\pmb {K}}_C$, can be obtained by solving Eqn. (4) by minimizing the $L^2$ norm least squares solution of $Ax = b$ (i.e., lsqminnorm function in MATLAB^28^), after which ${{\delta }\pmb {I}}_C$ can be calculated. The algorithm then updates the current eigenvalues, $\pmb {\lambda }_C$, by minimizing the norm of the difference, $\Vert \pmb {\lambda }_{C} - \pmb {\lambda }_{0} \Vert$, subject to constraints. The current inertia tensor, $\pmb {I}_C$, along with its eigendecomposition, $\pmb {\lambda }_{C}$ and $\pmb {E}_C$, are initialized using the raw marker data polluted with STAs. A step size, *s*, is initialized to be 1, and the current eigenvalue offset, ${\delta \pmb {\lambda }}_C$, is initialized to be $s\cdot (\pmb {\lambda }_0 - \pmb {\lambda }_C)$. After determining ${\delta \pmb {I}}_C$ from the solution to Eqn. (4), the current eigenvector offset, ${\delta \pmb {E}}_C$, is obtained utilizing Eqn. (5):

5 $$\begin{aligned} {{\delta }\pmb {e}}_{C,\;j} = \displaystyle \sum _{i=1,i\ne {j}}^{3} \frac{ {{\pmb {e}_{C,\;i}}^T} {{{\delta }\pmb {I}}_{C}} {\pmb {e}_{C,\;j}} }{{\lambda }_{C,\;j} - {\lambda }_{C,\;i}}{\pmb {e}_{C,\;i}} \end{aligned}$$

Next, the conditions described by Eqns. (6) and (7) are evaluated for each eigenvector:

6 $$\begin{aligned} \Vert {\pmb {e}_{C\;,\;j}}^T {{\delta }\pmb {e}}_{C\;,\;j}\Vert&< 10^{-7} \end{aligned}$$

7 $$\begin{aligned} \Vert (\pmb {e}_{C\;,\;j} + {{\delta }\pmb {e}}_{C\;,\;j}) - 1 \Vert&< 10^{-4} \end{aligned}$$

If these conditions are met, the estimates are updated. Otherwise, *s* and ${\delta \pmb {\lambda }}_C$ are halved and the process is repeated until $s < 2^{-13}$. If ${\Vert \pmb {\lambda }_{0} - \pmb {\lambda }_{C} \Vert < 10^{-7}}$ and the $\pmb {e}_{C,j}$ are mutually orthogonal, the final estimates are used to represent the current inertia tensor that has been appropriately denoised via perturbation theory.

Next, the task is to recover the mass distribution representing the “true” global marker positions, $\pmb {V}(t_k)=[\pmb {v}_{1}(t_k), \ldots , \pmb {v}_{N}(t_k)]$. Let $\pmb {I}_F(t)$ represent the denoised inertia tensor and $m_j$ represent the constant *non-unit* mass of the *j*th marker. At some time $t_k$, the relationship between the inertia tensor, marker positions, and mass distribution, $\pmb {m}=[m_{1}, \ldots , m_{N}]$, is given by Eqn. (8):

8 $$\begin{aligned} \pmb {I}_{F}(t_k) = f(\pmb {m},\;\pmb {V}(t_k)) = \begin{bmatrix} \displaystyle \sum _{j=1}^{N} m_j({y_{j}(t_k)}^2 + {z_{j}(t_k)}^2) &{} -\displaystyle \sum _{j=1}^{N} {m_j}x_{j}(t_k)y_{j}(t_k) &{} -\displaystyle \sum _{j=1}^{N} {m_j}x_{j}(t_k)z_{j}(t_k) \\ -\displaystyle \sum _{j=1}^{N} {m_j}x_{j}(t_k)y_{j}(t_k) &{} \displaystyle \sum _{j=1}^{N} {m_j}({x_{j}(t_k)}^2 + {z_{j}(t_k)}^2) &{} -\displaystyle \sum _{j=1}^{N} {m_j}y_{j}(t_k)z_{j}(t_k) \\ -\displaystyle \sum _{j=1}^{N} {m_j}x_{j}(t_k)z_{j}(t_k) &{} -\displaystyle \sum _{j=1}^{N} {m_j}y_{j}(t_k)z_{j}(t_k) &{} \displaystyle \sum _{j=1}^{N} {m_j}({x_{j}(t_k)}^2 + {y_{j}(t_k)}^2) \end{bmatrix} \end{aligned}$$

Eqn. (8) effectively contains 6 nonlinear equations at some time, $t_k$, as shown in Eqn. (9):

9 $$\begin{aligned} \begin{bmatrix} {{\pmb {I}_{F}}^{11}}(t_k) = f_{1}(\pmb {m},\;\pmb {V}(t_k)) \\ \vdots \\ {{\pmb {I}_{F}}^{33}}(t_k) = f_{6}(\pmb {m},\;\pmb {V}(t_k)) \end{bmatrix} \end{aligned}$$

Stacking equations for every time step yields $6N_{T}$ equations, where $N_{T}$ is the total number of time steps. The Levenberg-Marquardt algorithm^29^ can solve this system of nonlinear equations to obtain the non-unit mass distribution, $\pmb {m}$, which is then used to obtain the unconstrained center of mass (CM), $\pmb {T}_{UC}(t)$. The CM is then constrained with a scaling factor $\alpha$ such that the distance between the CM and the centroid (CM for unit mass distribution) is bound for each time instant (see "Appendix B" in the Supplementary Information). The constrained CM is given by Eqn. (10):

10 $$\begin{aligned} \pmb {T}_{C}(t) = (1-\alpha ) \cdot \pmb {c}(t) + \alpha \cdot \pmb {T}_{UC}(t) \end{aligned}$$

where $\pmb {c}(t)$ represents the centroid. Let $\Delta {\pmb {T}(t)}$ represent the offset of the constrained CM with respect to the centroid projected onto the first denoised eigenvector, $\pmb {e}_{F,1}$, given by Eqn. (11):

11 $$\begin{aligned} \Delta {\pmb {T}(t)} = {(\pmb {T}_{C}(t) - \pmb {c}(t))}^T \pmb {e}_{F,1} \end{aligned}$$

If $\Delta {\pmb {T}(t)}<0$ for the entire gait cycle, the constrained CM is treated as the final CM estimate, $\pmb {T}(t)$. However, if $\Delta {\pmb {T}(t)}>0$ for the entire gait cycle, then the constrained CM is reflected about the centroid to obtain the final CM estimate, $\pmb {T}(t)$. If $\Delta {\pmb {T}(t)}$ changes sign, the roots of $\Delta {\pmb {T}(t)}$ must be examined (see "Appendix C" in the Supplementary Information). Finally, the rotation matrix relating the body-fixed frame to the ground frame is estimated from $\pmb {T}(t)$ using the approach described by Wang et al.^30^.

### Simulation study

**Figure 1:**
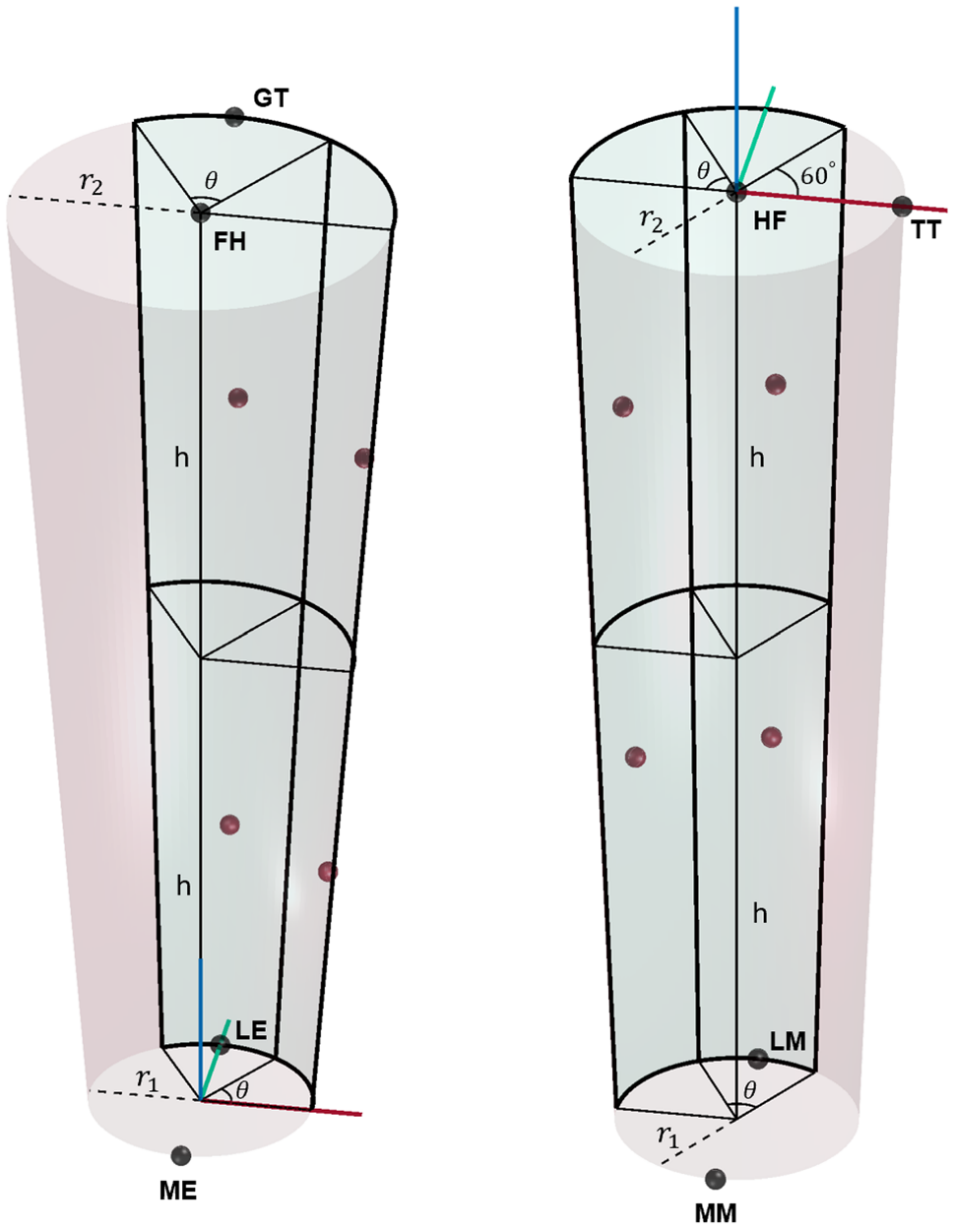
Conical frustums for the thigh (left) and the shank (right) with their respective reference marker configurations. Black spheres denote the anatomical landmarks (ME, LE, FH, GT, MM, LM, HF, TT). Red spheres denote the virtual skin-mounted markers, which can only be placed in the green shaded areas. The X (red), Y (green), Z (blue) vectors correspond to the anterior-posterior, medial-lateral, and inferior-superior directions, respectively. The inter-marker angular distance illustrated here is 60$^{\circ }$ and height difference is greater than 15 cm. The parameters ($r_{1},\;r_{2},\;h,\;\theta$) for the thigh are (5.5 cm, 9.5 cm, 25 cm, 60$^{\circ }$) and for the shank are (4 cm, 5.5 cm, 17.5 cm, 60$^{\circ }$).

The data utilized in this study was from an extensive open-source repository focused on providing researchers with the means to characterize STAs for a variety of activities and participants^31^. A conical frustum was created using a previously described anthropometric model^32^ (Fig. 1). Virtual marker configurations were generated based on locations for which STA data are available for a particular body segment^31,33^. Consistent with common marker sets^34,35^, the marker areas were divided into quadrants for which one virtual marker was placed randomly within each quadrant. A marker configuration was rejected if the markers were not adequately spaced. The result is a uniform distribution for *N* = 4 markers that is sufficient for estimating a body segment’s pose^36^. Following the virtual marker placement, anatomical landmarks are also generated for the thigh (medial epicondyle (ME), lateral epicondyle (LE), femoral head (FH), and greater trochanter (GT)) and shank (medial malleolus (MM), lateral malleolus (LM), fibular head (HF), and tibial tuberosity (TT)). A total of $N_{c} = 100$ configurations were generated.

Fortunately, STA and corresponding anatomical frame pose data has been made available for a variety of activities and subjects^31^. Here, treadmill walking data for 3 full gait cycles of the left leg for a 75 year old male participant was utilized^33^. Ground truth data are provided by two fluoroscopes configured to acquire X-ray images at a maximal frame rate^33^. First, the markers used in that data collection were assigned to a quadrant consistent with Fig. 1. For each quadrant, the STA data was obtained by averaging the raw STA data over all markers. Gait cycles were extracted from heel strikes, which was then used to temporally normalize both the ground truth and optical motion capture data. The ground truth data (collected at 30 Hz) was then interpolated such that the time stamps match that of the optical motion capture data (collected at 240 Hz). Finally, the STA data for the markers in a given quadrant on a given body segment were averaged for a characteristic STA profile for any virtual marker placed in that quadrant. The raw anatomical frame data provided by^33^ were also pre-processed to obtain a kinematic input to drive the model.

### Evaluation metrics

The quadrant specific STA noise models were applied to each marker configuration (denoted by *R*) to generate the noisy marker configuration data. The PCT, SVD-LS, and novel PCT-PT methods were used to obtain the reconstructed configurations (denoted by *r*). The reconstruction error for the $k^{th}$ configuration using the method, *M*, for the segment, *S*, was evaluated by the center of mass reconstruction offset (*TRO*), eigenvector reconstruction offset (*eRO*), and anatomical landmark reconstruction offset (*ALRO*), which are computed via Eqns. (12)-(14), respectively:

12 $$\begin{aligned} {TRO}_{k, M, S}(t)= & {} \Vert {T(t)}^R - {T(t)}^r \Vert \end{aligned}$$

13 $$\begin{aligned} {eRO}_{k, M, S, n}(t)= & {} {\text {cos}}^{-1} ({e_{n}(t)}^{R} \cdot {e_{n}(t)}^{r}),\;n=1,\;2,\;3 \end{aligned}$$

14 $$\begin{aligned} {ALRO}_{k, M, S, p}(t)= & {} \Vert {a_{p}(t)}^R - {a_{p}(t)}^r \Vert \end{aligned}$$

where $a_{p}(t)$ represents the global position vector for the $p^{th}$ anatomical landmark. For *eRO*, results will be presented for each eigenvector. Descriptive statistics (i.e., the mean and standard deviation) were computed for all proposed performance metrics. For the sake of completeness, the reconstructed anatomical frames^37^ were further evaluated with two additional performance metrics – the anatomical frame origin offset (*AFOO*) and the knee angle^38^ offset (*KAO*). The *AFOO* is a nonlinear combination of the errors of *TRO* and *eRO* for an *individual* body segment whereas the *KAO* is a nonlinear combination of the errors of *TRO* and *eRO* for *both* body segments.

## Results

Consistent with the findings of previous studies^19,25^, the PCT method produced discontinuous results for many of the generated virtual marker configurations. For the interested reader, "Appendix D" in the Supplementary Information describes how these discontinuities in the PCT results are a direct consequence of how the method was developed mathematically. Thus, only the subset of marker configurations that produced continuous time series were used to calculate descriptive statistics for the PCT method to capture what the method is capable of when physiologically consistent results are produced. Specifically, the number of continuous configurations for the PCT method were 23 and 17 for the thigh and shank, respectively. For the results to follow, the average was taken over all marker configurations for the SVD-LS and PCT-PT methods while the average was taken over the marker configurations that produced valid results for the PCT method.

**Figure 2:**
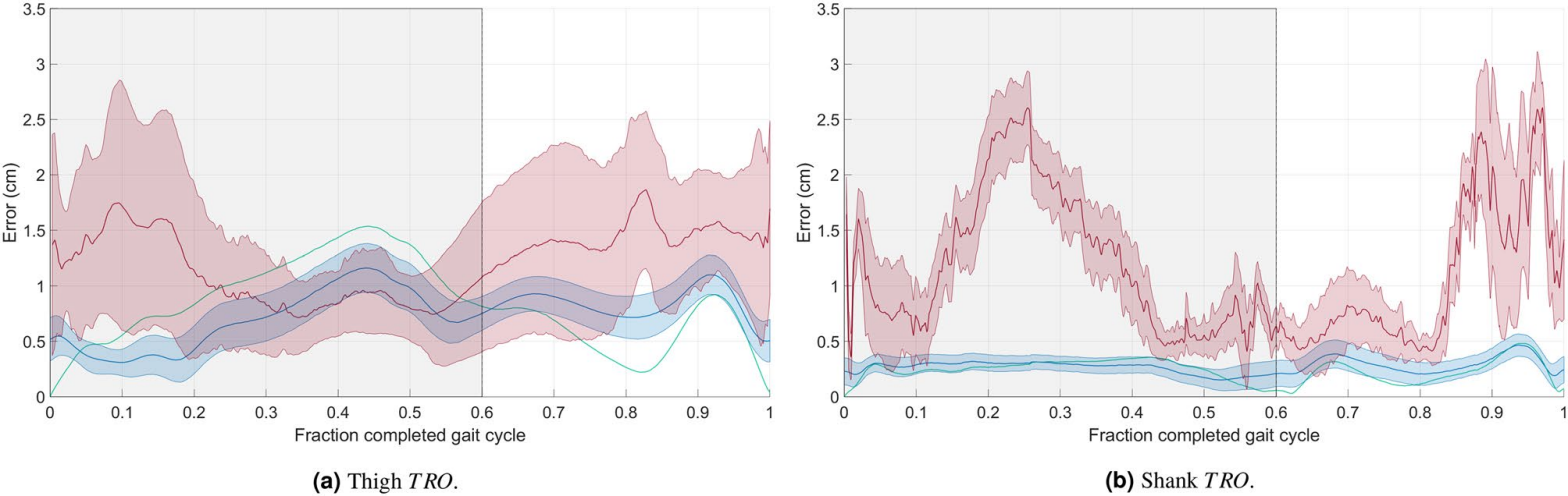
Center of Mass reconstruction offset (*TRO*) for the (**a**) thigh and (**b**) shank over the entire gait cycle. The average *TRO* line (solid) and standard deviation envelope (shaded) is shown for the PCT (red), PCT-PT (blue), and SVD-LS (green). The shaded window represents the stance phase where the vertical black line bounding the shaded window on the right corresponds to toe-off.

The SVD-LS center of mass reconstruction offset (*TRO*) results did not have an envelope for either body segment. This finding suggests that the *TRO* for the SVD-LS method is *independent* of which marker configuration was used. As is illustrated in Fig. 2, the envelope for the PCT-PT method fell below the SVD-LS curve for the thigh and was comparable to SVD-LS curve for the shank during majority of the stance phase. For the majority of the gait cycle, the PCT-PT method also fell below the average curve for the PCT method for both body segments. Unsurprisingly given the inconsistency in the results, the PCT results had the largest and least smooth envelope for both the thigh and shank.

**Figure 3:**
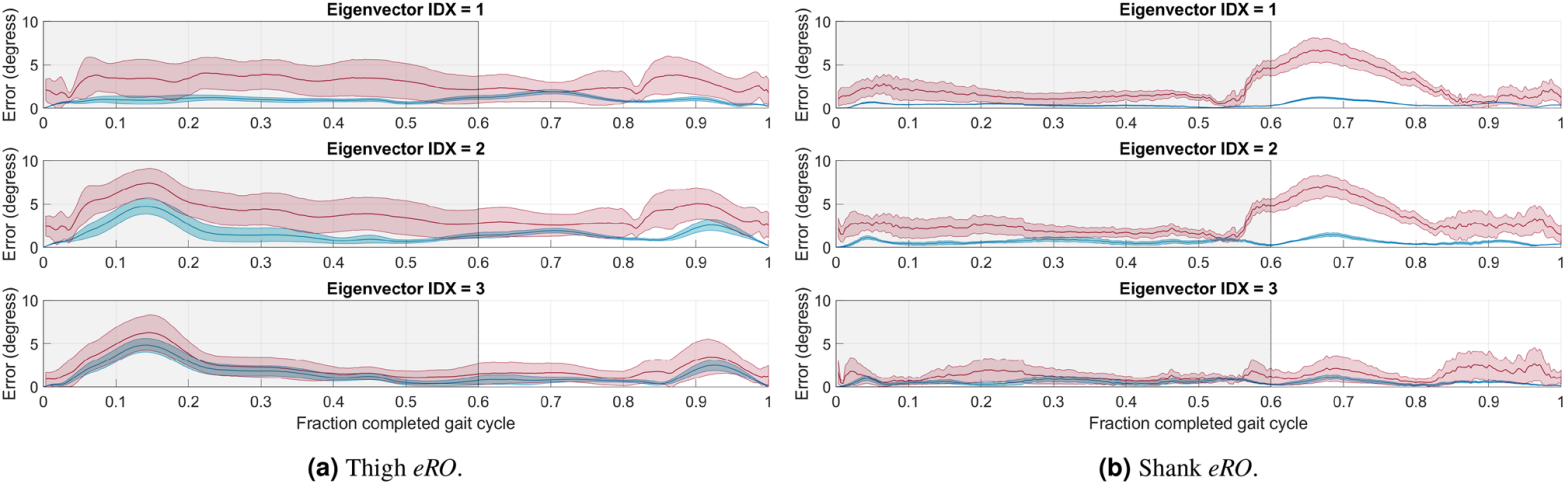
Eigenvector Reconstruction Offset (*eRO*) for the (**a**) thigh and (**b**) shank over the entire gait cycle. The average *eRO* line (solid) and standard deviation envelope (shaded) is shown for the PCT (red), PCT-PT (blue), and SVD-LS (green) for the first eigenvector (top), second eigenvector (center), and third eigenvector (bottom). The shaded window represents the stance phase where the vertical black line bounding the shaded window on the right corresponds to toe-off.

Next, the SVD-LS and PCT-PT eigenvector reconstruction offset (*eRO*) results were identical. The *eRO* results for these two methods were the smallest for the first eigenvector for the thigh and the smallest for the third eigenvector for the shank as is illustrated in Fig. 3. Note that the offsets for the other two eigenvectors for both body segments were also small (i.e., less than 5 degrees). The average eigenvector offset curve for the PCT-PT method is below the average eigenvector offset curve for the PCT for both body segments. The envelopes for the first and second eigenvectors also have minimal overlap during the majority of the stance phase for the thigh and during the majority of the gait cycle for the shank.

**Figure 4:**
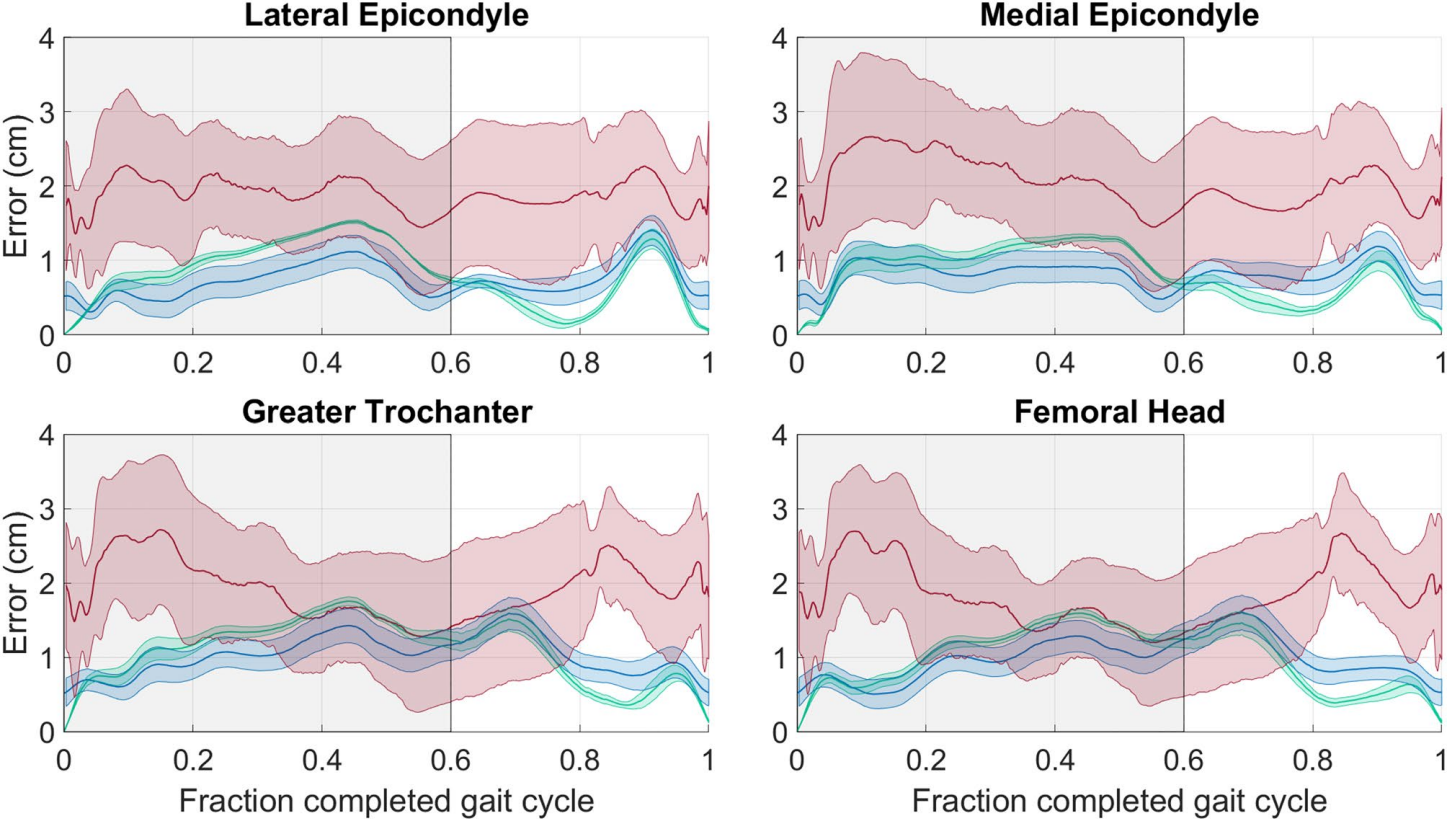
Anatomical Landmark Reconstruction Offset (*ALRO*) for the thigh over the entire gait cycle. The subplots illustrate the results of the four anatomical markers corresponding to the thigh. The average *CPRO* line (solid) and standard deviation envelope (shaded) is shown for the PCT (red), PCT-PT (blue), and SVD-LS (green). The shaded window represents the stance phase where the vertical black line bounding the shaded window on the right corresponds to toe-off.

**Figure 5:**
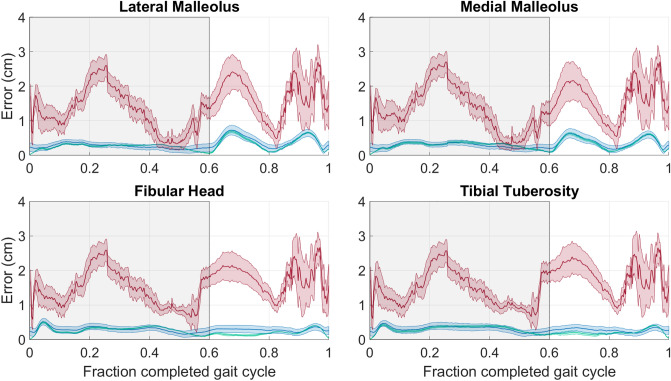
Anatomical Landmark Reconstruction Offset (*ALRO*) for the shank over the entire gait cycle. The subplots illustrate the results of the four anatomical markers corresponding to the shank. The average line (solid) and standard deviation envelope (shaded) is shown for the PCT (red), PCT-PT (blue), and SVD-LS (green). The shaded window represents the stance phase where the vertical black line bounding the shaded window on the right corresponds to toe-off.

Finally, the anatomical landmark reconstruction offset (*ALRO*) for the SVD-LS method had an envelope that differed from the *TRO* results for the thigh (Fig. 4) and shank (Fig. 5). The average curve for the PCT-PT method fell below the envelope of the SVD-LS method during the majority of the stance phase for the thigh and was comparable to the envelope of the SVD-LS method for the shank. For both body segments, the envelope for the PCT-PT method was below the average curve for the PCT during majority of the gait cycle. For the thigh (shank), the envelopes for all three methods overlap less (more) frequently for the epicondyles (malleoli) than the other two anatomical landmarks. The results for all performance metrics are reported in Tables 1 and 2 for the thigh and shank, respectively.

**Table 1:** Performance metrics for each algorithm for the thigh.

		PCT			PCT-PT			SVD-LS		
		GC	StP	SwP	GC	StP	SwP	GC	StP	SwP
*TRO* [cm]		1.21 ± 0.63	1.07 ± 0.58	1.43 ± 0.71	0.75 ± 0.18	0.69 ± 0.19	0.84 ± 0.17	0.82 ± 0.00	0.98 ± 0.00	0.58 ± 0.00
*eRO* [deg]	1	2.9 ± 1.7	3.2 ± 1.8	2.5 ± 1.6	1.0 ± 0.2	0.9 ± 0.2	1.1 ± 0.2	1.0 ± 0.2	0.9 ± 0.2	1.1 ± 0.2
	2	3.9 ± 1.7	4.3 ± 1.8	3.3 ± 1.6	1.6 ± 0.5	1.7 ± 0.6	1.5 ± 0.3	1.6 ± 0.5	1.7 ± 0.6	1.5 ± 0.3
	3	2.3 ± 1.2	2.3 ± 1.2	1.8 ± 1.2	1.5 ± 0.4	1.8 ± 0.5	1.0 ± 0.3	1.5 ± 0.4	1.8 ± 0.5	1.0 ± 0.3
*ALRO* [cm]	LE	1.89 ± 0.84	1.90 ± 0.81	1.87 ±0.88	0.74 ± 0.18	0.71 ± 0.20	0.79 ± 0.16	0.83 ± 0.06	1.01 ± 0.04	0.56 ± 0.08
	ME	2.04 ± 0.89	2.14 ± 0.89	1.88 ± 0.88	0.82 ± 0.20	0.82 ± 0.21	0.82 ± 0.18	0.82 ± 0.09	1.00 ± 0.09	0.55 ± 0.09
	GT	1.91 ± 0.88	1.90 ± 0.88	1.91 ± 0.89	1.03 ± 0.20	1.01 ± 0.21	1.06 ± 0.20	1.09 ± 0.09	1.24 ± 0.08	0.86 ± 0.10
	FH	1.83 ± 0.80	1.78 ± 0.80	1.90 ± 0.81	0.99 ± 0.20	0.93 ± 0.20	1.07 ± 0.19	0.99 ± 0.08	1.11 ± 0.07	0.82 ± 0.10

The mean and standard deviations are calculated across all marker configurations for the full gait cycle (GC), stance phase (StP), and swing phase (SwP).

**Table 2:** Performance metrics for each algorithm for the shank.

		PCT			PCT-PT			SVD-LS		
		GC	StP	SwP	GC	StP	SwP	GC	StP	SwP
*TRO* [cm]		1.14 ± 0.33	1.20 ± 0.31	1.06 ± 0.38	0.28 ± 0.09	0.26 ± 0.08	0.30 ± 0.11	0.24 ± 0.00	0.25 ± 0.00	0.22 ± 0.00
*eRO* [deg]	1	2.4 ± 0.8	1.7 ± 0.7	3.4 ± 0.9	0.4 ± 0.1	0.3 ± 0.1	0.6 ± 0.1	0.4 ± 0.1	0.3 ± 0.1	0.6 ± 0.1
	2	2.9 ± 0.9	2.2 ± 0.8	4.1 ± 1.1	0.6 ± 0.2	0.6 ± 0.2	0.6 ± 0.1	0.6 ± 0.2	0.6 ± 0.2	0.6 ± 0.1
	3	1.3 ± 0.9	1.1 ± 0.8	1.5 ± 1.1	0.5 ± 0.2	0.6 ± 0.2	0.5 ± 0.1	0.5 ± 0.2	0.6 ± 0.2	0.5 ± 0.1
*ALRO* [cm]	LM	1.44 ± 0.36	1.28 ± 0.30	1.69 ± 0.44	0.33 ± 0.09	0.26 ± 0.08	0.43 ± 0.11	0.30 ± 0.01	0.24 ± 0.01	0.37 ± 0.02
	MM	1.45 ± 0.37	1.33 ± 0.30	1.63 ± 0.46	0.32 ± 0.09	0.28 ± 0.08	0.39 ± 0.11	0.31 ± 0.02	0.28 ± 0.02	0.36 ± 0.02
	FH	1.56 ± 0.33	1.42 ± 0.28	1.78 ± 0.41	0.29 ± 0.09	0.29 ± 0.09	0.28 ± 0.10	0.24 ± 0.01	0.28 ± 0.01	0.19 ± 0.02
	TT	1.60 ± 0.33	1.43 ± 0.28	1.84 ± 0.41	0.30 ± 0.10	0.32 ± 0.10	0.28 ± 0.10	0.26 ± 0.02	0.30 ± 0.02	0.20 ± 0.02

The mean and standard deviations are calculated across all marker configurations for the full gait cycle (GC), stance phase (StP), and swing phase (SwP).

**Figure 6:**
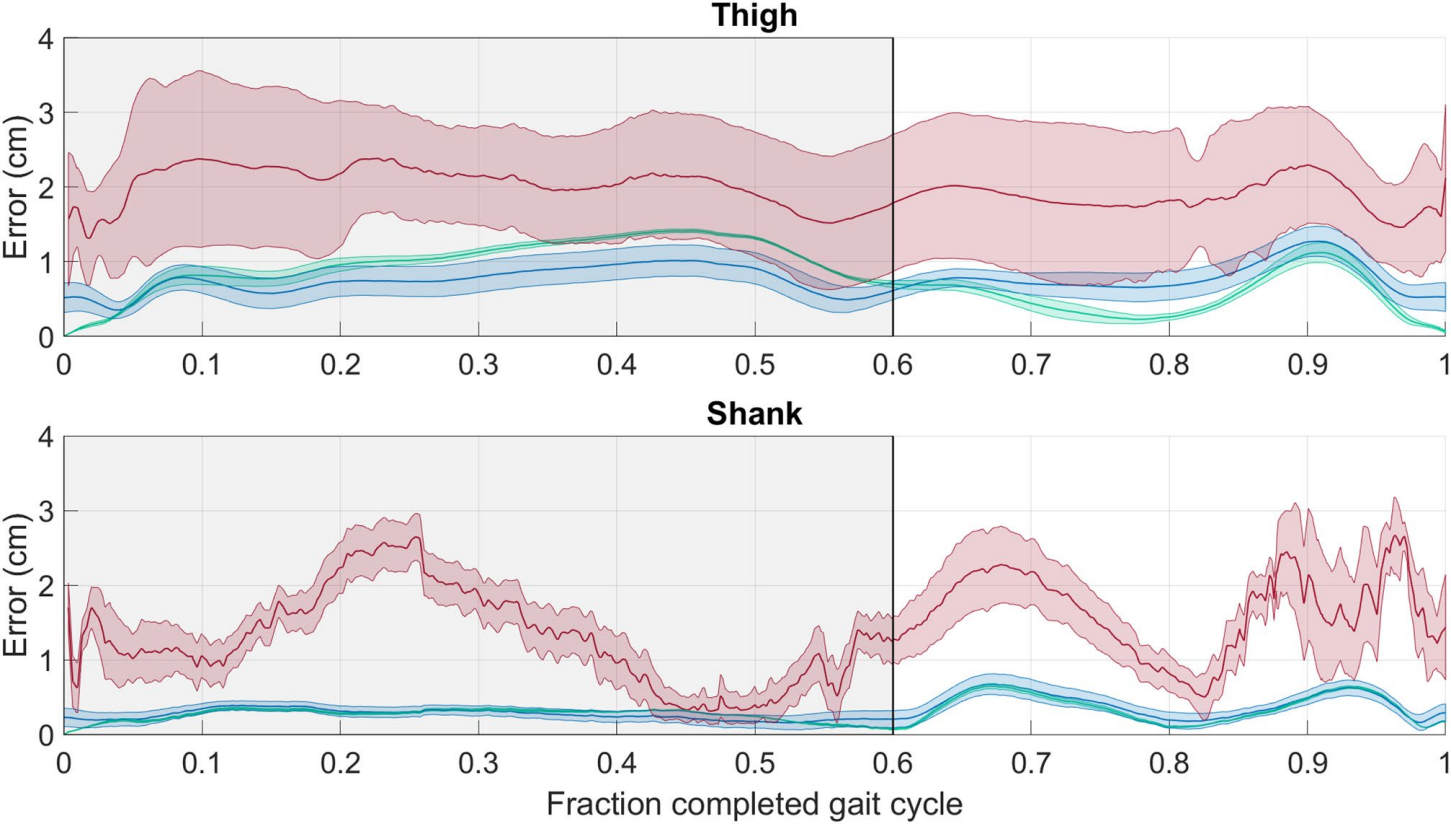
Anatomical Frame origin offset (*AFOO*) over the entire gait cycle. The average line (solid) and standard deviation envelope (shaded) is shown for the PCT (red), PCT-PT (blue), and SVD-LS (green) for the thigh (top) and shank (bottom). The shaded window represents the stance phase where the vertical black line bounding the shaded window on the right corresponds to toe-off.

For the anatomical frame origin offset (*AFOO*), the envelope for the PCT-PT method was below the envelope for the SVD-LS method during majority of the stance phase for the thigh and during the majority of the entire gait cycle for the shank (Fig. 6). The envelope for the PCT-PT method was also below the envelope for the PCT method for the majority of the gait cycle. The PCT method had the largest envelope while the average curve had the largest offset for both body segments.

**Figure 7:**
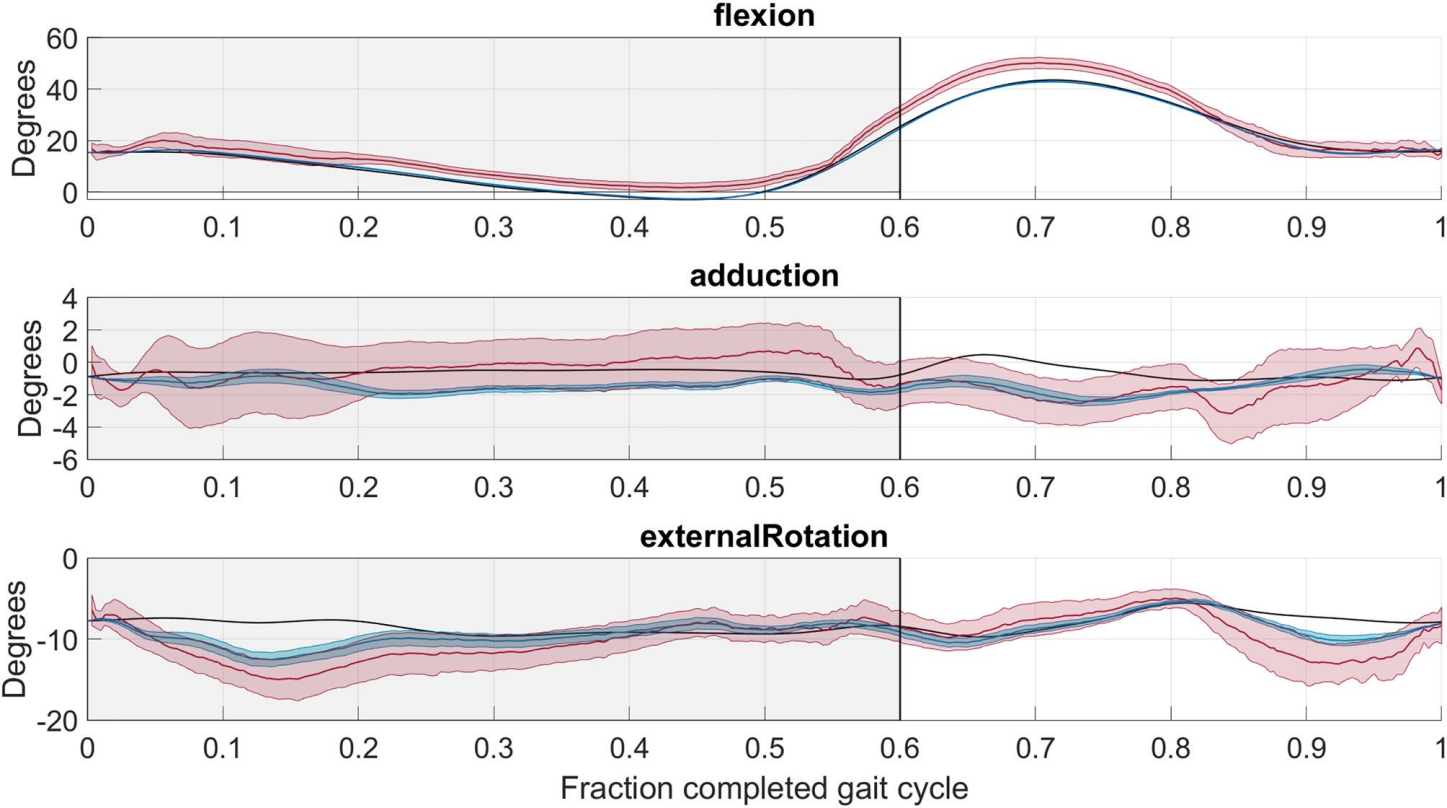
Knee Angle offset (*KAO*) over the entire gait cycle. The ground truth curves are plotted as solid black lines. The knee angles shown above are Flexion-Extension (top), Abduction-Adduction (center), and Internal-External Rotation (bottom). The average line (solid) and standard deviation envelope (shaded) is shown for the PCT (red), PCT-PT (blue), and SVD-LS (green). The shaded window represents the stance phase where the vertical black line bounding the shaded window on the right corresponds to toe-off.

For all knee joint angles, the reconstructed values for the PCT-PT and SVD-LS methods applied to virtual marker trajectories with representative STA data agree well with the ground truth curve for majority of the gait cycle (Fig. 7). For flexion-extension, both methods have excellent agreement for the entire gait cycle. By contrast, the PCT method overestimates flexion-extension, deviates more from the ground truth curve for internal-external rotation, and exhibits larger envelopes across all three angles.

## Discussion

This study provides additional evidence that the PCT method is significantly limited by the underlying mathematical constraints governing its optimization process^15,19,25^. As a direct result of these constraints^15^, this method frequently yields physiologically meaningless results (i.e., discontinuities), specifically following the mass redistribution optimization. As such, the performance of the PCT method was shown to vary significantly depending on the marker configuration used, which is consistent with previous studies^19,25^. Recall the PCT method yielded continuous results for only 23 marker configurations for the thigh and 17 marker configurations for the shank. The novel PCT-PT method significantly improves upon the PCT method by producing physiologically meaningful results for all marker configurations that are consistently superior on average with smaller envelopes.

Surprisingly, the SVD-LS center of mass offset (*TRO*) results did not have an envelope (i.e., standard deviations equal to zero in Tables 1 and 2), implying that the *TRO* for this method is independent of the marker configuration. It is likely that this finding is a consequence for using the same quadrant-specific representative STA data for the virtual markers, which is also likely artificially inflating the performance of the algorithms presented here as compared to results reported in the literature^33,39,40^. However, this lack of variance was not true for the SVD-LS eigenvector reconstruction offset (*eRO*) results. Interestingly, the eigenvector estimates for the PCT-PT and SVD-LS methods were identical. The eigenvectors for the PCT-PT method are obtained by solving a least-squares fitting problem after translating the global frame to the center of mass^30^. The SVD-LS method^16^ essentially solves a least-squares problem to obtain the centroid (center of mass with unit mass distribution) and the eigenvectors simultaneously. The fact that the PCT-PT and SVD-LS methods yield the same rotation matrix estimates implies that the least-squares solution for the rotation matrix is also independent of the center of mass estimates.

While the SVD-LS center of mass results did not have an envelope, the anatomical marker reconstruction offsets (*ALRO*) did have envelopes since they combine the *TRO* and *eRO*, the latter of which did vary across marker configurations. For markers that are relatively far from the center of mass (e.g., the femoral head for the thigh and the malleoli for the shank), the *ARLO* envelopes for these distant markers differ from those of their closer counterparts for both the SVD-LS and PCT-PT methods. Although there is greater overlap between the two methods’ envelopes for the distant markers, the average *ARLO* envelope for the PCT-PT method lies below that of the SVD-LS method during majority of the stance phase for the thigh. For the thigh, the PCT-PT method provides superior marker reconstruction than the SVD-LS method during majority of the stance phase. For the shank segment, the PCT-PT method provides comparable marker reconstruction to the SVD-LS with low errors for all markers during the entire gait cycle. It is reasonable that the PCT-PT method does not outperform the SVD-LS method since the shank has much lower STA than the thigh^9^, the SVD-LS method shows high attenuation of instrumental noise^8^, and the PCT-PT method is designed to minimize STA. For the interested reader, the *ARLO* results are also consistent with the virtual marker reconstruction offsets as well, the results of which are included in "Appendix E" in the Supplementary Information.

The knee angle results for the PCT-PT and SVD-LS methods are also identical. This finding is a direct result of the knee angle estimates solely depending on the axes of the femoral and tibial anatomical frames, which solely depend on the eigenvectors of each body segments’ frames. The results for PCT-PT and SVD-LS applied to virtual marker trajectories with representative STA data have excellent agreement with the ground truth curve for flexion-extension while having better agreement than the PCT for the other two knee angles. This finding is unsurprising given that flexion-extension estimates are typically very robust to noise^10,14,41^. For all knee angles, the PCT-PT and SVD-LS methods produce estimates that consider all marker configurations and have a smaller envelope size, making them both superior to the PCT method The results for the anatomical frame origin offset for the thigh segment show that the PCT-PT method outperforms the SVD-LS method during the stance phase and outperforms the PCT method during the gait cycle. For the shank, the results for the SVD-LS and PCT-PT methods are comparable, but both outperformed the PCT method.

### Limitations and future work

A notable limitation is only utilizing STA data from a single subject^31^. Additionally, the STA data for each body segment was averaged for a specific quadrant for the virtual markers, which reduces the resolution for marker-specific STA trends. Future work should consider STA data collections with the same experimental set up^33^ and data reporting^31^ with participants with varying age, sex, and anthropometry. This study investigated marker placements useful for effectively applying the PCT-PT method. Future work could explore the possibility of developing a simulation tool capable of handling any marker configuration by translating it to the conical frustrum models utilized by the PCT-PT method. With respect to marker configurations, other future work could investigate how the PCT-PT method performs on widely used marker configurations like the Helen-Hayes marker set^34^ and Vicon plug-in gait model^35^.

It should also be noted that SVD-LS, PCT, and PCT-PT are all methods designed to eliminate the non-rigid body components of STA. Previous work^10^ has shown that these methods do not improve upon each other (i.e., applying multiple non-rigid body methods does not mitigate any rigid body STA). As such, future work should also consider how to mitigate *rigid body* components of STA, which have been shown to be related to joint kinematics^17^.

## Conclusion

The novel PCT-PT method has been shown to have superior performance compared to the PCT method from which it builds. This study also provides a detailed description regarding why the PCT method so frequently produces discontinuous (i.e., physiologically impossible) results. For position-based metrics, it has also been shown to produce better reconstruction estimates than the SVD-LS method for the thigh during majority of the stance phase and produce comparable estimates for the shank during the entire gait cycle during treadmill walking. It has also been shown to produce the same eigenvectors and consequently, knee angle estimates as the SVD-LS method. Future work should consider application of this method for non-uniform marker placements, collection of varied STA data and application of the method developed here to that data.

### Supplementary Information

Supplementary Information.

## Data Availability

The datasets analysed during the current study are available in repository created by Cereatti et al.^31^.
