# Supplementary material for: A simulation study to investigate an extension to the point cluster technique
Source: Sci Rep. 2023 Nov 15;13:19941. doi: 10.1038/s41598-023-47144-2 (PMC10651841; doi:10.1038/s41598-023-47144-2)
Supplement: Supplementary file 1 — Supplementary Information. [file 41598_2023_47144_MOESM1_ESM.pdf]

Supplementary Information

Appendix

A Obtaining the Linear System of Equations for the Perturbation Matrix

The inertia tensor is symmetric, which means the perturbation matrix $\delta \boldsymbol{I}_C$ was defined as follows:

$$\delta \boldsymbol{I}_C = \begin{bmatrix} k_1 & k_2 & k_3 \\ k_2 & k_4 & k_5 \\ k_3 & k_5 & k_6 \end{bmatrix} \tag{1}$$

The change in the jth eigenvalue can be computed from perturbation theory[1,2] using the following equation:

$$\delta \lambda_{C,j} = \boldsymbol{e}_{C,j}{}^T \delta \boldsymbol{I}_C \boldsymbol{e}_{C,j} = \begin{bmatrix} e_{C,j}{}^x & e_{C,j}{}^y & e_{C,j}{}^z \end{bmatrix} \begin{bmatrix} k_1 & k_2 & k_3 \\ k_2 & k_4 & k_5 \\ k_3 & k_5 & k_6 \end{bmatrix} \begin{bmatrix} e_{C,j}{}^x \\ e_{C,j}{}^y \\ e_{C,j}{}^z \end{bmatrix} \tag{2}$$

Multiplying the terms in Eqn. 2 yields:

$$\delta \lambda_{C,j} = \begin{bmatrix} (e_{C,j}{}^x)^2 & 2e_{C,j}{}^x e_{C,j}{}^y & 2e_{C,j}{}^x e_{C,j}{}^z & (e_{C,j}{}^y)^2 & 2e_{C,j}{}^y e_{C,j}{}^z & (e_{C,j}{}^z)^2 \end{bmatrix} \begin{bmatrix} k_1 \\ k_2 \\ k_3 \\ k_4 \\ k_5 \\ k_6 \end{bmatrix} \tag{3}$$

Applying the results in Eqn. 3 to each eigenvalue and rearranging gives:

$$\begin{bmatrix} (e_{C,1}{}^x)^2 & 2e_{C,1}{}^x e_{C,1}{}^y & 2e_{C,1}{}^x e_{C,1}{}^z & (e_{C,1}{}^y)^2 & 2e_{C,1}{}^y e_{C,1}{}^z & (e_{C,1}{}^z)^2 \\ (e_{C,2}{}^x)^2 & 2e_{C,2}{}^x e_{C,2}{}^y & 2e_{C,2}{}^x e_{C,2}{}^z & (e_{C,2}{}^y)^2 & 2e_{C,2}{}^y e_{C,2}{}^z & (e_{C,2}{}^z)^2 \\ (e_{C,3}{}^x)^2 & 2e_{C,3}{}^x e_{C,3}{}^y & 2e_{C,3}{}^x e_{C,3}{}^z & (e_{C,3}{}^y)^2 & 2e_{C,3}{}^y e_{C,3}{}^z & (e_{C,3}{}^z)^2 \end{bmatrix} \begin{bmatrix} k_1 \\ k_2 \\ k_3 \\ k_4 \\ k_5 \\ k_6 \end{bmatrix} = \begin{bmatrix} \delta \lambda_{C,1} \\ \delta \lambda_{C,2} \\ \delta \lambda_{C,3} \end{bmatrix} \tag{4}$$

Equation 4 can be written more compactly as a linear system of equations:

$$\boldsymbol{A}_C \cdot \delta \boldsymbol{K}_C = \delta \boldsymbol{\lambda}_C \tag{5}$$

B Deriving a Bound for Constraining the Center of Mass

Let t denote the fraction completed of gait cycle (i.e., $t \in [0,1]$). The distance between the center of mass (CM) and centroid (CM for unit mass distribution) is bound using the function defined as follows:

$$D_{max}(t) = \begin{cases} a_0 + bt & 0 \leq t \leq 0.5 \\ a_1 - bt & 0.5 < t \leq 1 \end{cases} \tag{6}$$

The values used were $a_0 = 0.7, a_1 = 2.9$ and $b = 2.2$ respectively. This choice was made so that the above function also acted as an upper bound for the norm of the centroid of the noise model at each time step.

C Estimating the Final Center of Mass Via Root Analysis

Let $\boldsymbol{T}_R(t)$ denote the reflected center of mass (CM), which is obtained from the following equation:

$$\boldsymbol{T}_R(t) = \begin{cases} 2\boldsymbol{c}(t) - \boldsymbol{T}_C(t) & \text{if } \Delta \boldsymbol{T}(t) > 0 \\ \boldsymbol{T}_C(t) & \text{if } \Delta \boldsymbol{T}(t) \leq 0 \end{cases} \tag{7}$$

If $\Delta\boldsymbol{T}(t)$ does not change sign during the gait cycle, the final CM estimate $\boldsymbol{T}(t)$ is given by Eqn. 7. However, if $\Delta\boldsymbol{T}(t)$ does change signs during the gait cycle, the roots of $\Delta\boldsymbol{T}(t)$ must be considered. A change in sign will cause $\boldsymbol{T}_R(t)$ to be discontinuous and yield non-physiological results.

Let t_u denote the root of $\Delta\boldsymbol{T}(t)$ with index u such that $u \in [1, 2, \ldots, N_r]$ where N_r is the number of roots of $\Delta T(t)$. Two possible cases are considered here. In the first case, the function switches sign from positive to negative at first root of the function for $\Delta\boldsymbol{T}(t)$. Contingent upon this condition being met, the values of $\boldsymbol{T}_R(t)$ would then be set equal to $2\boldsymbol{c}(t) - \boldsymbol{T}_C(t)$ for all time instants between that root and the next root. In the second case, the function switches sign from negative to positive at first root of the function for $\Delta\boldsymbol{T}(t)$. Here, the values of $\boldsymbol{T}_R(t)$ are set equal to $\boldsymbol{T}_C(t)$ for all time instants between that root and the next root. In either case, the time series $\boldsymbol{T}_R(t)$ corresponding to the reflected CM is now continuous and is used as the final estimate for $\boldsymbol{T}(t)$.

D Discontinuities in the Point Cluster Technique

The point cluster technique[3] (PCT) utilizes the following mass redistribution function, $m(\cdot)$, to minimize the effects of soft tissue artifacts (STAs) that produce erroneous non-rigid body motion:

$$m(\varepsilon(t_s))_i = \varepsilon(t_s) - \frac{\Delta L(t_s)_i}{\max(\Delta L(t_s)_i)}\ ,\ i = 1\ ,\ \ldots\ ,\ N \tag{8}$$

where t_s is the current time step, $\Delta L(\cdot)_i$ is the magnitude of the displacement of the ith marker relative to some local reference (e.g., a static calibration), and ε is the single parameter to be solved for through optimization (e.g., the Levenberg-Marquardt technique[4]).

From Eqn. 8, there are two possible cases that result in discontinuous marker results (i.e., physiologically meaningless results). In the first case, the optimization parameter, $\varepsilon(t)$, is a discontinuous function of time, which will also lead to discontinuous marker mass. Any subsequent calculations of marker results either preserves or amplifies the magnitude of these discontinuities. A representative example of this case is illustrated in Fig. S1.

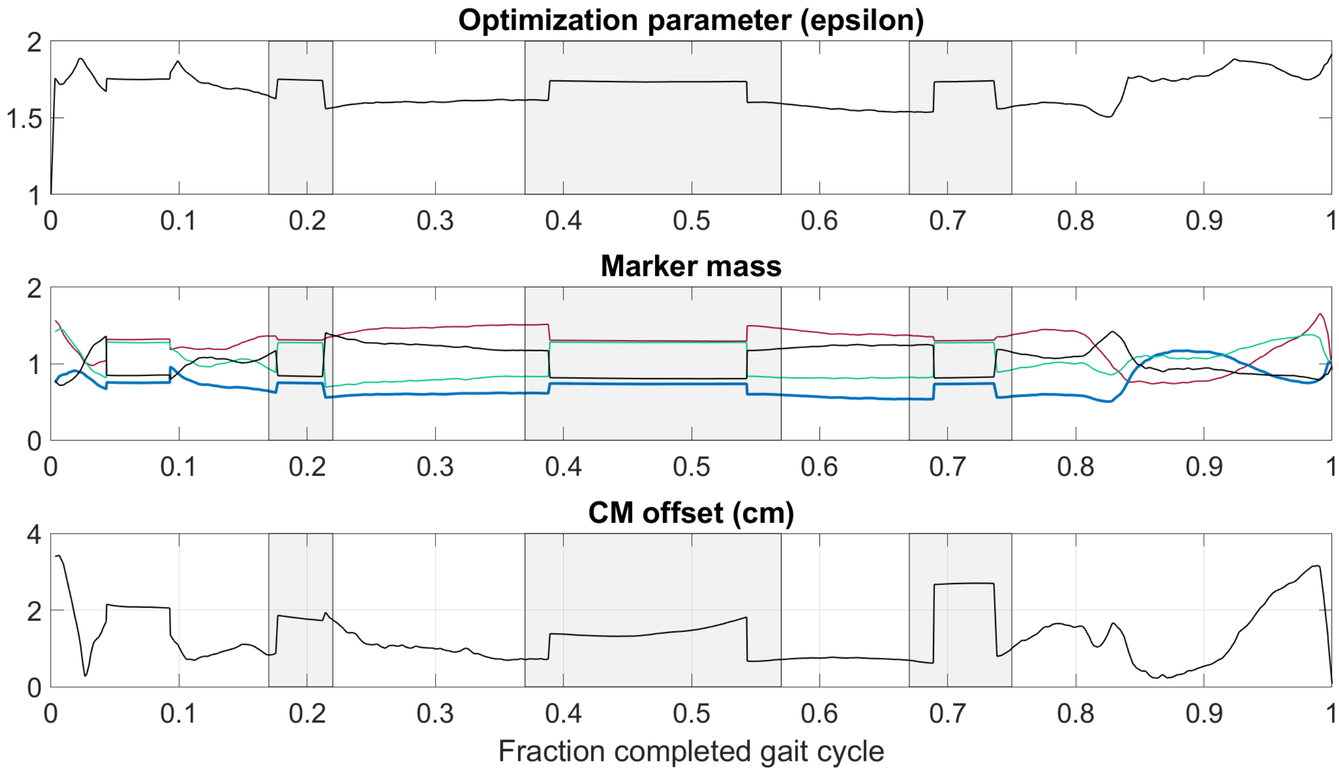

Figure S1. Example of discontinuous marker results for PCT due to optimization parameters discontinuities. The plots for the following quantities have been shown above: $\varepsilon(t)$ (top), marker masses (center) and center of mass (CM) offset (referred to as TRO in the main body of the paper) (bottom). The legend for the markers are: m_1 (red), m_2 (blue), m_3 (green) and m_4 (black). The discontinuities occur in the shaded regions.

In the second case, the optimization parameter, ε, is "practically" continuous, meaning sufficiently small changes between time steps, while the marker ratio is discontinuous. Consider the case where the ratio defined in the second term on the right hand side of Eqn. 8 is discontinuous at t_s while changes in ε from t_s to t_{s+1} are sufficiently small. Let marker j have the largest local displacement at the time instant t_s as described here:

$$\arg\max_i (\Delta L(t_s)_i) = j \tag{9}$$

In this case, the mass for marker j at time instant t_s is given per:

$$m(\varepsilon(t_s))_j = \varepsilon(t_s) - 1 \tag{10}$$

Suppose marker j no longer has the largest local displacement at the next time step, t_{s+1}. Importantly, this result would mean that Eqn. 10 no longer holds at t_{s+1}. For the mass of marker j to be a continuous function of time, the condition described next must be satisfied:

$$\|m(\varepsilon(t_{s+1}))_j - (\varepsilon(t_{s+1}) - 1)\| < \delta \tag{11}$$

where δ represents a sufficiently small value such that the change in ε from t_s to t_{s+1} is small enough for the parameter to be considered essentially continuous. Substituting Eqn. 8 evaluated at t_{s+1} into the inequality described in Eqn. 11 yields the following:

$$\left\| 1 - \frac{\Delta L(t_{s+1})_j}{\max(\Delta L(t_{s+1})_i)} \right\| < \delta \tag{12}$$

Since the aforementioned ratio equals unity at t_s and is discontinuous at t_s, the inequality described in Eqn. 12 does not hold. Therefore, the mass of marker j will be discontinuous. The computation of marker results preserves the magnitude of these discontinuities. An example plot of a discontinuous time series for PCT has been shown below in Fig. S2.

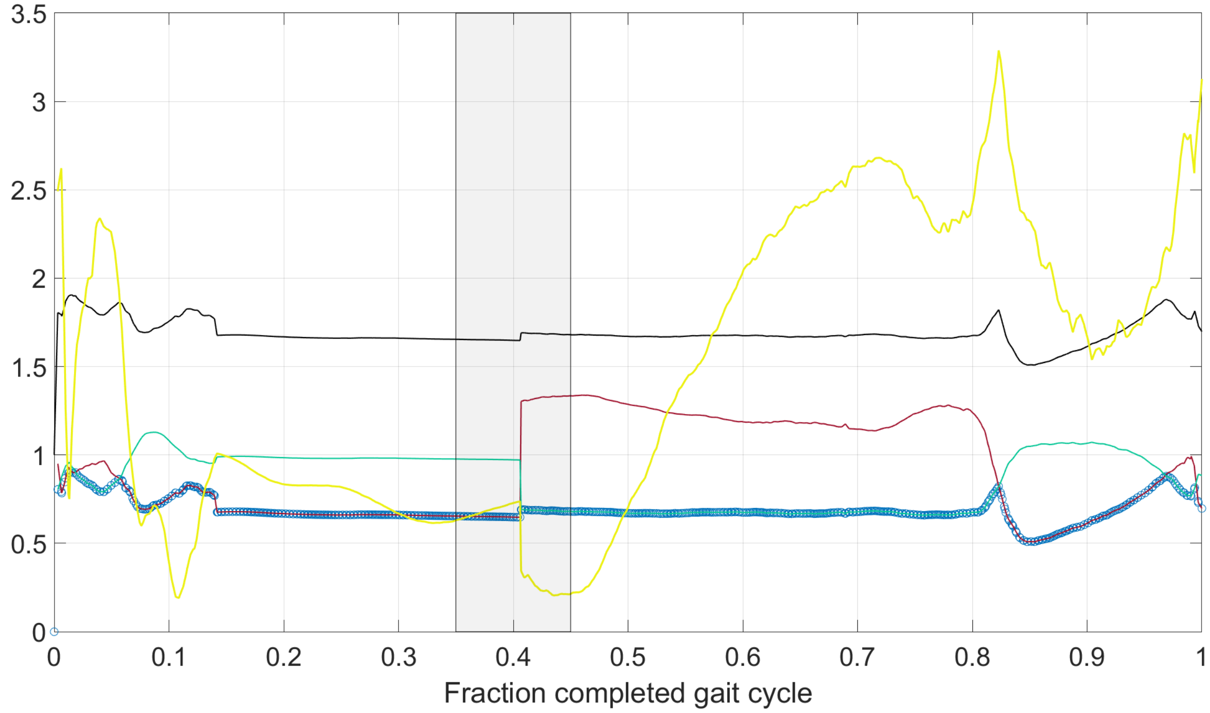

Figure S2. Example of discontinuous marker results for PCT due to marker ratio discontinuities. The plots for the following quantities have been shown above: $\varepsilon(t)$ (black), $\varepsilon(t) - 1$ (blue circle), $m_2(t)$ (red), $m_4(t)$ (green), $TRO(t)$ (yellow). The discontinuity in marker results occurs in the shaded region.

E Cluster Point Reconstruction Offset Results

The cluster point reconstruction error ($CPRO$) for the kth configuration with method, M, for segment, S, was evaluated per:

$$CPRO_{k,M,S,j}(t) = \|v_j(t)^R - v_j(t)^r\| \tag{13}$$

where $v_j(t)^R$ denotes the actual virtual marker position and $v_j(t)^r$ denotes the reconstructed virtual marker position. For both body segments, the $CPRO$ for the SVD-LS method had an envelope that differed from its corresponding center of mass offset (TRO) results. The results are illustrated in Fig. S3 for the thigh and in Fig. S4 for the shank.

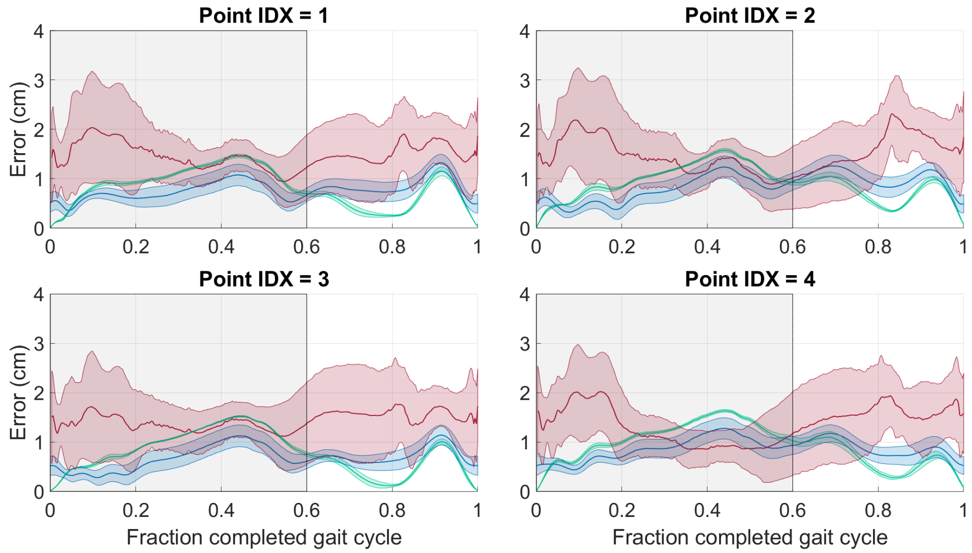

Figure S3. Cluster Point Reconstruction Offset *CPRO* for the thigh over the entire gait cycle. The subplots illustrate the results of the four virtual markers placed on the thigh. The average *CPRO* line (solid) and corresponding standard deviation envelope (shaded) is shown for the PCT (red), PCT-PT (blue), and SVD-LS (green). The shaded window represents the stance phase where the vertical black line bounding the shaded window on the right corresponds to toe-off.

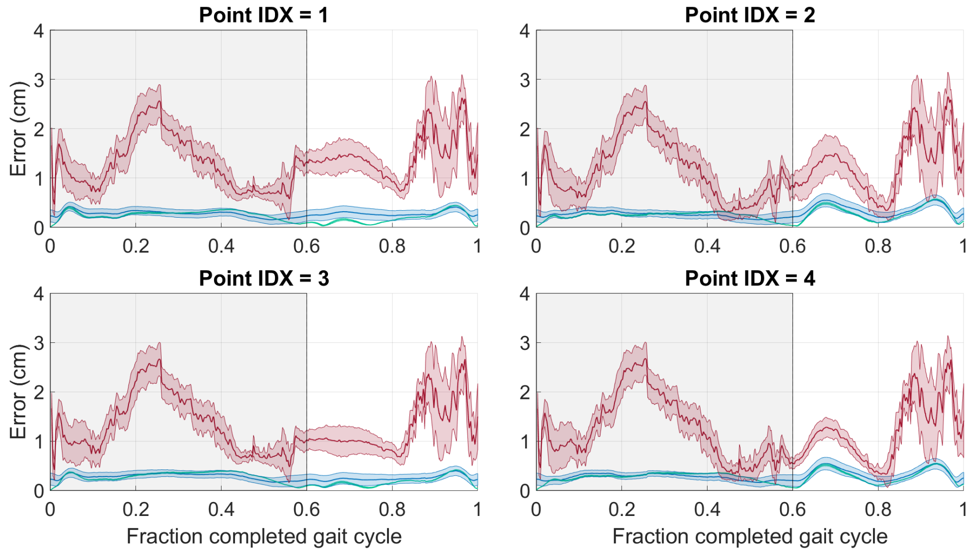

Figure S4. Cluster Point Reconstruction Offset *CPRO* for the shank over the entire gait cycle. The subplots illustrate the results of the four virtual markers placed on the shank. The average *CPRO* line (solid) and corresponding standard deviation envelope (shaded) is shown for the PCT (red), PCT-PT (blue), and SVD-LS (green). The shaded window represents the stance phase where the vertical black line bounding the shaded window on the right corresponds to toe-off.

The envelope for the PCT-PT method fell below the SVD-LS envelope for the majority of the stance phase for the thigh and for the majority of the entire gait cycle for the shank. The envelope for the PCT-PT method was below the average curve for the PCT during majority of the gait cycle for both body segments. The PCT had the largest envelope with time series are not as smooth as those from the PCT-PT and SVD-LS results for both body segments. Tables S1 and S2 contain the results for each virtual marker for each method.

Table S1. Cluster Point Reconstruction Offset (*CPRO*) for each algorithm for the thigh. The mean and standard deviations are calculated across all marker configurations for the full gait cycle (GC), stance phase (StP), and swing phase (SwP).

		PCT			PCT-PT			SVD-LS		
		GC	StP	SwP	GC	StP	SwP	GC	StP	SwP
CPRO [cm]	1	1.48 ±0.66	1.45 ± 0.61	1.52 ± 0.72	0.77 ± 0.18	0.72 ± 0.18	0.85 ± 0.18	0.81 ± 0.04	0.99 ± 0.03	0.55 ± 0.05
	2	1.49 ± 0.66	1.42 ± 0.65	1.58 ± 0.68	0.85 ± 0.19	0.75 ± 0.19	1.01 ± 0.19	0.90 ± 0.04	1.01 ± 0.03	0.74 ± 0.06
	3	1.43 ± 0.69	1.35 ± 0.63	1.56 ± 0.79	0.69 ± 0.18	0.66 ± 0.20	0.73 ± 0.17	0.78 ± 0.03	0.97 ± 0.02	0.49 ± 0.05
	4	1.37 ± 0.66	1.24 ± 0.58	1.57 ± 0.79	0.87 ± 0.18	0.86 ± 0.18	0.88 ± 0.18	0.93 ± 0.05	1.11 ± 0.03	0.67 ± 0.07

Table S2. Cluster Point Reconstruction Offset (*CPRO*) for each algorithm for the shank. The mean and standard deviations are calculated across all marker configurations for the full gait cycle (GC), stance phase (StP), and swing phase (SwP).

		PCT			PCT-PT			SVD-LS		
		GC	StP	SwP	GC	StP	SwP	GC	StP	SwP
CPRO [cm]	1	1.35 ± 0.32	1.29 ± 0.29	1.45 ± 0.37	0.28 ± 0.09	0.27 ± 0.09	0.28 ± 0.10	0.22 ± 0.01	0.25 ± 0.01	0.18 ± 0.01
	2	1.22 ± 0.36	1.17 ± 0.32	1.29 ± 0.42	0.30 ± 0.09	0.25 ± 0.08	0.36 ± 0.11	0.26 ± 0.01	0.23 ± 0.01	0.29 ± 0.01
	3	1.30 ± 0.32	1.32 ± 0.29	1.28 ± 0.37	0.27 ± 0.09	0.28 ± 0.08	0.25 ± 0.10	0.24 ± 0.01	0.28 ± 0.01	0.18 ± 0.01
	4	1.22 ± 0.31	1.24 ± 0.30	1.20 ± 0.33	0.30 ± 0.09	0.27 ± 0.08	0.34 ± 0.10	0.28 ± 0.01	0.27 ± 0.01	0.29 ± 0.01

References

1. Sakurai, J. J. & Commins, E. D. Modern quantum mechanics, revised edition (1995).

2. Landau, L. D. & Lifshitz, E. M. *Quantum mechanics: non-relativistic theory*, vol. 3 (Elsevier, 2013).

3. Andriacchi, T. P., Alexander, E. J., Toney, M., Dyrby, C. & Sum, J. a. A point cluster method for in vivo motion analysis: applied to a study of knee kinematics. *J. biomechanical engineering* **120**, 743–749 (1998).

4. Moré, J. J. The levenberg-marquardt algorithm: implementation and theory. In *Numerical analysis*, 105–116 (Springer, 1978).
